# Depuration and Starvation Regulate Metabolism and Improve Flesh Quality of Yellow Catfish (*Pelteobagrus fulvidraco*)

**DOI:** 10.3390/metabo13111137

**Published:** 2023-11-08

**Authors:** Ya Zhou, Yang Xiong, Xianlin He, Xiaoshu Xue, Guo Tang, Jie Mei

**Affiliations:** 1 College of Fisheries, Huazhong Agricultural University, Wuhan 430070, China; asian_chou@outlook.com; 2 College of Animal Science and Technology, Chongqing Three Gorges Vocational College, Chongqing 404155, China; hexianlin@cqsxzy.edu.cn (X.H.); shya@cqsxzy.edu.cn (X.X.); tangguo@cqsxzy.edu.cn (G.T.)

**Keywords:** depuration, starvation, catfish, flesh quality, metabolomics

## Abstract

Fat deposition and off-flavor in the muscle are the main problems affecting flesh quality in aquaculture fish, especially in catfish, leading to low acceptability and reduced market price. Yellow catfish is an important aquaculture fish in China. In this study, 40 days of depuration and starvation treatment were explored to improve the muscle quality of aquaculture yellow catfish. After depuration and starvation, the body weight, condition factor (CF) and mesenteric fat index (MFI) were all significantly decreased 20 days after treatment. The metabolomic profiles in muscle were characterized to analyze the muscle quality in yellow catfish. The results showed that the content of ADP, AMP, IMP, glutamic acid and taurine were significantly increased between 20 and 40 days post-treatment in the muscle of yellow catfish during the treatment, which was positively associated with the flesh tenderness and quality. In contrast, aldehydes and ketones associated with off-flavors and corticosterone associated with bitter taste were all decreased at 20 days post-treatment. Considering the balance of body weight loss and flesh quality improvement, depuration and starvation for around 20 days is suitable for aquaculture yellow catfish. Our study not only provides an effective method to improve the flesh quality of aquaculture yellow catfish but also reveals the potential mechanism in this process.

## 1. Introduction

Fish is an important source of high-quality protein for human, while flavor, appearance and texture are the primary criteria for evaluating the quality of the fish fresh from the commercial market [1,2,3]. Pond culture is the main mode for freshwater aquaculture. However, the physiological condition of fish and flesh quality are greatly affected by the pond culture environment [4,5]. With the intensification of pond aquaculture, off-flavor in the flesh of cultured fish species, especially in catfish, has become a major problem that leads to poor acceptability and reduced market price [6,7,8].

To improve the flesh quality of fish cultured in ponds, depuration and starvation have been confirmed to be effective techniques. Recent studies have shown that a certain period of starvation can improve the flesh quality and flavor in crucian carp (*Carassius auratus*), dentex (*Dentex dentex*), Atlantic salmon (*Salmo salar*) and common carp (*Cyprinus carpio* L.) [9,10,11,12], while depuration with micro-flowing water can also improve the muscle quality and taste of crucian carp and grass carp (*Ctenopharyngodon idella*) [13,14]. In addition, some other studies showed that depuration and starvation could been used to improve fish flesh in some fish species, including grass carp, southern flounder (*Paralichthys lethostigma*), Silver Carp (*Hypophthalmichthys molitrix*) and Nile tilapia (*Oreochromis niloticus*) [15,16,17,18]. However, the mechanism of depuration and starvation treatment to enhance muscle mass have not been clarified.

Yellow catfish (*Pelteobagrus fulvidraco*) is an important aquaculture fish in Asian, with high nutrient vale and no intramuscular bones [19]. The aquaculture of yellow catfish has rapidly grown over the last decade in China, and the annual yield increased from 217 thousand tons in 2011 to 587 thousand tons in 2021 due to the development of breeding technology and high-density cultivation mode. However, high fish densities and low water exchange rates usually cause excessive fat deposition and off-flavors and reduce the taste of yellow catfish [20,21]. In the present study, depuration and starvation were performed to improve the flesh quality of cultured yellow catfish. The ultra-performance liquid chromatography-quadrupole-time of flight-mass spectrometry (UPLC-QTOF/MS) based on untargeted metabolomics was used to investigate the muscle metabolites of yellow catfish during depuration and starvation treatment. Our results provide a theoretical basis and technical reference for improving the muscle quality of cultured fish species.

## 2. Materials and Methods

### 2.1. Experimental Design and Ethics Statement

**Figure 1:**
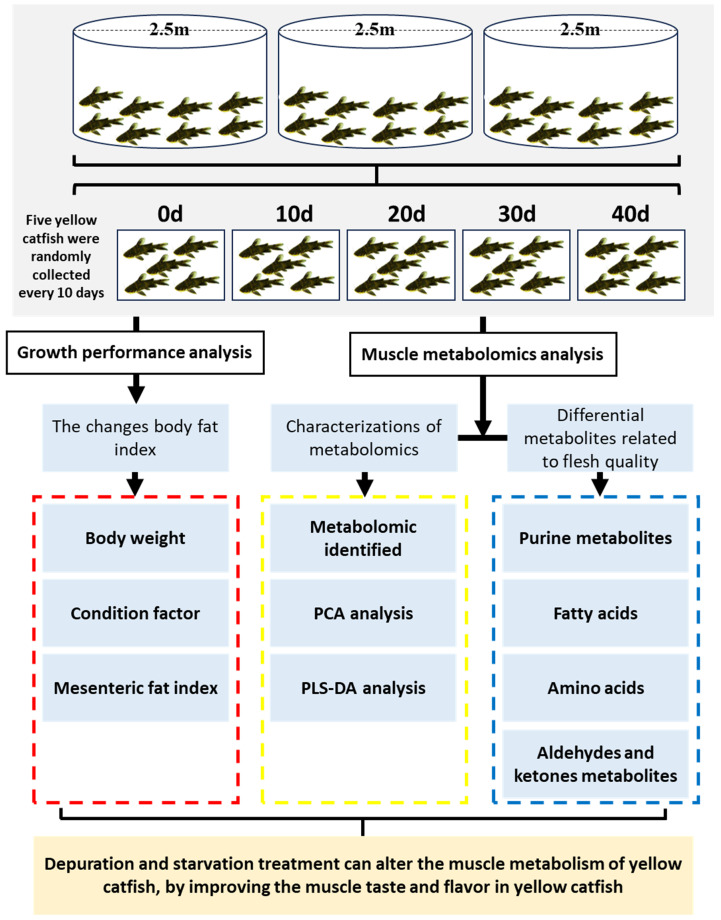
Experimental workflow for the depuration and starvation in yellow catfish.

All-male yellow catfish (71.45 ± 6.21 g) were collected from the same culture pond in FuXiang Aquaculture company (Wanzhou, Chongqing, China) and transported into three circular pools (2.5 m diameter × 1.0 m water deep, triplicates) with a micro-flowing water depuration system. There were 830 individuals of yellow catfish placed in each pool in which the water exchange velocity was kept at 0.8 L/s. During the depuration period, all fish were deprived of food. Based on the previous report [9], dissolved oxygen (DO), pH and temperature were maintained between 7.3 ± 0.5 mg/L, 7.9 ± 0.2 and 22.5 ± 1.5 °C. Nine yellow catfish were randomly collected from three pools (three from each pool) every 10 days (a:0 d, b:10 d, c:20 d, d:30 d, e:40 d), among which five fish were randomly chosen and anesthetized with 200 mg/L MS-222, after which we measured the body length, weight, and mesenteric fat. The experimental design is shown in Figure 1. The condition factor (CF) and mesenteric fat index (MFI) were measured by the following method: CF (%) = bodyweight (g)/bodylength^3^ (cm)^3^ × 100%, MFI (%) = weight of mesenteric fat (g)/bodyweight (g) × 100%.

All animal procedures were authorized by the Animal Care and Use Committee of Huazhong Agricultural University and carried out in accordance with the Guidelines for Care and Use of Laboratory Animals of Huazhong Agricultural University.

### 2.2. Metabolite Extraction

Metabolite extraction was performed according to previous studies [13]. Briefly, muscle samples (~50 mg) were frozen in the ultra-cold freezer (−80 °C). After thawing, samples were transferred in an Eppendorf (EP) tube and added with 800 μL ice-cold methanol and 10 μL 2-chlorophenylalanine (2.8 mg/mL). All muscle samples were broken by a tissue crusher at 60 HZ for 5 min. After a 15 min centrifugation at 12,000× *g* and 4 °C, the samples were filtered using a 0.22 um membrane filter after extraction, and then 200 μL supernatant was transferred to the sample vials for further detection. 

### 2.3. UPLC-Triple-TOF /MS Analysis

Chromatographic separations were carried out using the ultra-performance liquid chromatography (UPLC) system (SCIEX, UK). An ACQUITY UPLC T3 column (100 mm × 2.1 mm, 1.8µm, Waters, UK) was used for the reversed-phase separation. The mobile phase consisted of solvent A (water, 0.1% formic acid), and solvent B (acetonitrile, 0.1% formic acid) was introduced for the metabolite’s separation. The gradient elution conditions were the following with a flow rate of 0.4 mL/min: 5% solvent B for 0–0.5 min, 5–100% solvent B for 0.5–7 min, 100% solvent B for 7–8 min, 100–5% solvent B for 8–8.1 min, and 5% solvent B for 8.1–10 min. The column temperature was maintained at 35 °C. 

The TripleTOF 5600 Plus system (SCIEX, Warrington, UK) was used to detect metabolites eluted from the column under the following conditions: curtain air (N_2_) pressure at 30 PSI, ion source gas1 and gas2 at 60 PSI, ion spray floating voltage at 5 kV under positive-ion (ES+) mode and −4.5 kV under negative-ion (ES-) mode, heater temperature at 650 °C. The MS data were acquired in the IDA mode. The TOF mass range was 60–1200 *m*/*z* with a scan time of 0.15 s.

### 2.4. Metabolomics Data Processing 

The pretreatment of acquired liquid chromatography-mass spectrometry (LC-MS) data was performed using XCMS software (version 3.9.3). Raw data files were converted into mzXML format and then processed using the XCMS, while CAMERA and metaX toolbox were implemented with R software (R version R3.4.3). Each ion was identified by the comprehensive information of retention time and *m*/*z*. The intensity of each peak was recorded, and a three-dimensional matrix containing arbitrarily assigned peak indices (retention time-*m*/*z* pairs), sample names (observations) and ion intensity information (variables) was generated. Then, the information was matched to the in-house and public database. The open access databases, KEGG and HMDB, were used to annotate the metabolites by matching the exact molecular mass data (*m*/*z*) to those from the database within a threshold of 10 ppm. The peak intensity data were further preprocessed using metaX. Features detected in <50% of QC (quality control) samples or 80% of test samples were removed, and values for missing peaks were extrapolated with the k-nearest neighbor algorithm to further improve the data quality. PCA was performed to detect outliers and batch effects using the preprocessed dataset. QC-based robust LOESS signal correction was fitted to the QC data with respect to the order of injection to minimize signal intensity drift over time. In addition, the relevant standard deviations (SD) of the metabolic features were calculated across all QC samples, and samples with SD > 30% were removed. The data groups were then standardized before analysis. Data normalization was performed on all samples using the probabilistic quotient normalization algorithm. Then, QC-robust spline batch correction was performed using QC samples. The *p* value was analyzed by Student’s *t*-tests, which was then adjusted for multiple tests using an FDR (Benjamini–Hochberg) for the different metabolite selection. The supervised PLS-DA (Partial Least Squares–Discriminant Analysis) was also conducted using metaX for variables and the discriminant profiling statistical method to identify more specific differences between the groups. The cut-off of variable important in projection (VIP) was set as 1.0 to choose important features.

### 2.5. Statistical Analysis

One-way analysis of variance (ANOVA) was used for evaluating the significant differences using Statistical Package for Social Sciences (SPSS) program software version 25.0 (Chicago, IL, USA). *p* < 0.05 was set as the significance threshold.

## 3. Results

### 3.1. The Changes of Body Weight and Body Fat Index after Depuration and Starvation in Yellow Catfish

**Figure 2:**
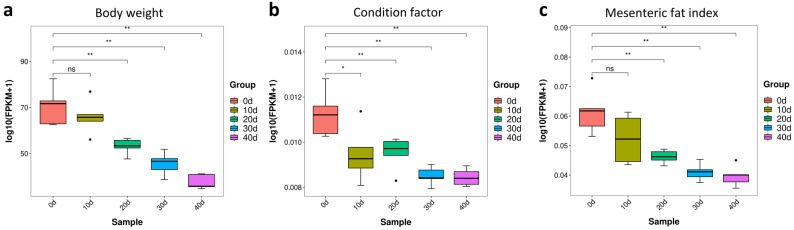
The growth performance and body fat index in yellow catfish after depuration and starvation. (**a**) Body weight; (**b**) condition factor (CF); (**c**) mesenteric fat index (MFI). CF (%) = bod − weight (g)/bodylength^3^ (cm)^3^ × 100%; MFI (%) = weight of mesenteric fat (g)/bodyweight (g) × 100%. Note: ns, no significance, the * and ** represent significant differences between two groups (*p* < 0.05, *p* < 0.01).

To improve the muscle quality of yellow catfish, depuration and starvation were performed with relatively constant water parameters and food deprivation for 0, 10, 20, 30 or 40 days. Comparison to the groups of 0 day treatment, the body weight was not significantly changed at 10 days post-treatment, but it had significantly decreased at 20, 30 and 40 days post-treatment (Figure 2a). After depuration and starvation, the condition factor (CF) and mesenteric fat index (MFI) were significantly decreased at 10 and 20 days post-treatment, respectively (Figure 2b,c).

### 3.2. Characterizations of Metabolomics in the Muscle of Yellow Catfish during the Process of Depuration and Starvation

The metabolomic profiles of muscle in yellow catfish were investigated to examine the muscle quality of yellow catfish. According to the peak heights in the spectra, 550 metabolites (ES + 265, ES − 285) were identified in the muscle samples of yellow catfish during depuration, as detailed in https://ngdc.cncb.ac.cn/omix/release/OMIX005160 (accessed on 30 October 2023). Based on *m*/*z* vales, 303 molecular features were annotated by comparing with the standard libraries (HMDB, Human Metabolome Database and LMPD, Lipid Maps Proteome Database), including 179 lipids, 40 amino acids and amino acid derivatives, 23 nucleic acid hydrolysates and their derivatives, 16 carbohydrates and carbohydrate conjugates, and 45 other chemicals.

**Figure 3:**
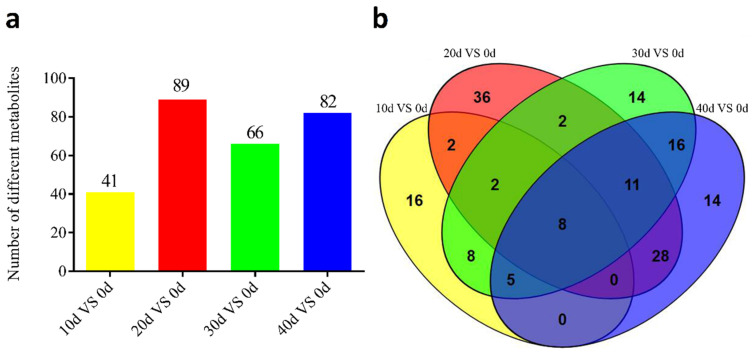
The identification of different metabolites in five groups. (**a**) The number of differential metabolites in comparison groups; (**b**) Venn analysis of differential metabolites in comparison groups.

Based on the determination criteria (VIP > 1.5, *p* < 0.05) of the OPLS-DA model, 162 differentially expressed metabolites were identified in the muscles of yellow catfish between the control group and purified groups under different treatment times, while 58 representative metabolites with significantly differential expression are shown in Table 1. These metabolites are mainly related to amino acid metabolism, fat metabolism and purine metabolism. The metabolic numbers for four different comparisons (10 d vs. 0 d, 20 d vs. 0 d, 30 d vs. 0 d, and 40 d vs. 0 d) were 41, 89, 66 and 82 of different metabolites, respectively (Figure 3a). Furthermore, the number of unique metabolites in the 20 d vs. 0 d was slightly higher than in the other three comparison groups (10 d vs. 0 d, 30 d vs. 0 d, and 40 d vs. 0 d). There were eight metabolites shared in four groups during the process of depuration and starvation treatment (Figure 3b). The content of AMP increased at 10–20 days and decreased at 20–40 days, while the content of histamine was consistently reduced during the depuration and starvation.

**Figure 4:**
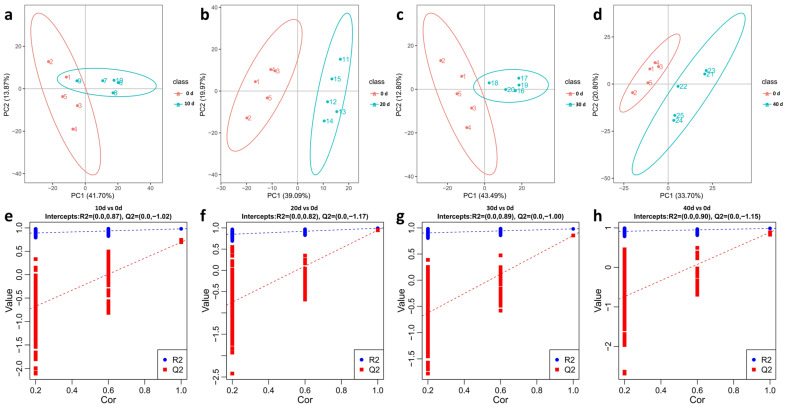
The PCA and PLS-DA analysis of yellow catfish muscle in the five groups. PCA: (**a**) (10 d vs. 0 d); (**b**) (20 d vs. 0 d); (**c**) (30 d vs. 0 d); (**d**) (40 d vs. 0 d). PLS-DA: (**e**) (10 d vs. 0 d); (**f**) (20 d vs. 0 d); (**g**) (30 d vs. 0 d); (**h**) (40 d vs. 0 d).

Principal component analysis and PLS-DA analysis were performed on the identified metabolites to investigate the trend of separation among the groups during the process of depuration and starvation treatment. The PCA models of 10 d vs. 0 d, 20 d vs. 0 d, 30 d vs. 0 d, and 40 d vs. 0 d were carried out and are displayed in Figure 4a (PC1: 41.70%; PC2: 13.87%), 4B (PC1: 39.09%; PC2: 19.97%), 4C (PC1: 43.49%; PC2: 12.80%), 4D (PC1: 33.70%; PC2: 20.80%). The PLS-DA models were also performed by making comparisons between 10 d vs. 0 d, 20 d vs. 0 d, 30 d vs. 0 d, and 40 d vs. 0 d, and they are illustrated in Figure 4e–h, respectively. Meanwhile, the PLS-DA analysis showed that the Q2 values were all less than 0, indicating significant differences in muscle metabolites within the five groups (*p* < 0.05). These results suggest that depuration and starvation treatment could induce significant changes in the muscle metabolomics profile in yellow catfish.

### 3.3. Differential Metabolites Related to Flesh Quality

#### 3.3.1. Purine Metabolites Changes in Yellow Catfish Muscle during Depuration and Starvation

**Figure 5:**
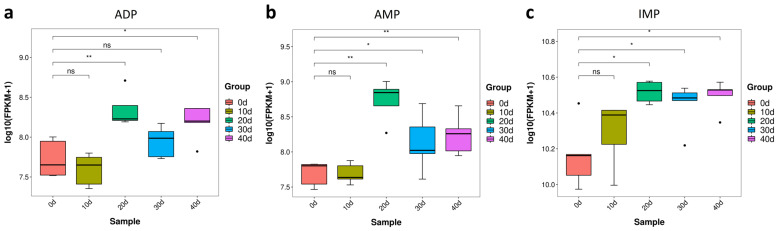
Purine metabolites content of yellow catfish muscle in five groups. (**a**) ADP; (**b**) AMP; (**c**) IMP. note: ns, no significance, the * and ** represent significant differences observed between two groups (*p* < 0.05, *p* < 0.01).

Adenosine diphosphate (ADP), adenosine 5′-monophosphate (AMP) and inosine-5′-monophosphate (IMP) are the degradation production of ATP and associated with the flesh tenderness and quality [13]. The contents of ADP, AMP and IMP in the muscle of yellow catfish were analyzed during the processes of depuration and starvation. As shown in Figure 5, the contents of ADP and AMP significantly increased at 20 days post-treatment (*p* < 0.05) and decreased at 30 days post-treatment (Figure 5a,b). In addition, the content of IMP significantly increased at 20 days post-treatment (*p* < 0.05) and was not obviously changed between 20 and 40 days post-treatment (Figure 5c). These data indicate that depuration and starvation for 20 days is the suitable condition to improve the contents of ADP, AMP and IMP.

#### 3.3.2. The Changes of Fatty Acids in Yellow Catfish Muscle during Depuration and Starvation

**Figure 6:**
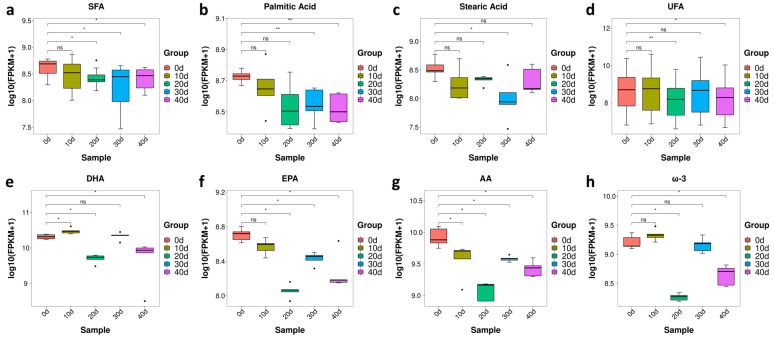
Fatty acid content of yellow catfish muscle in five groups. (**a**) SFA; (**b**) C16:0; (**c**) C18:0; (**d**) UFA. (**e**) DHA; (**f**) EPA; (**g**) AA; (**h**) ω-3. note: ns, no significance, the * and** represent significant differences observed between two groups (*p* < 0.05, *p* < 0.01).

Excessive fat deposition negatively affects the flesh quality in aquaculture [22], which usually occurs in yellow catfish [23]. Since the mesenteric fat index (MFI) was significantly decreased after depuration and starvation treatment (Figure 2c), we further checked the contents of fatty acids and their metabolites. There was a significant decrease in the contents of saturated fatty acids (SFAs) in yellow catfish muscle after 20 days post-treatment (Figure 6a). The contents of palmitic acid (C16:0) and stearic acid (C18:0), two of the most commonly consumed saturated fatty acids, were gradually reduced after depuration and starvation treatment and significantly reduced at 30 days post-treatment (Figure 6b,c). There was a slight decrease in the content of unsaturated fatty acids (UFA), while a significant decrease in UFA was observed at 20 and 40 days post-treatment (Figure 6d). Compared with the control, the contents of docosahexaenoic acid (DHA), eicosapentaenoic acid (EPA), arachidonic acid (AA) and eicosatrienoic acid (ω-3) were lower between 20 and 40 days post-treatment, while all have the lowest expression at 20 days post-treatment (Figure 6e–h). In brief, depuration and starvation treatment could reduce the index of fat and fatty acids in yellow catfish.

#### 3.3.3. The Changes of Amino Acids in Yellow Catfish Muscle during Depuration and Starvation

**Figure 7:**
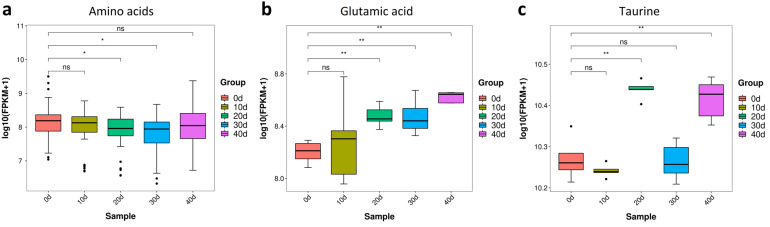
Amino acid content of yellow catfish muscle in five groups. (**a**) Amino acids; (**b**) glutamic acid; (**c**) taurine. note: ns, no significance, the * and ** represent significant differences observed between two groups (*p* < 0.05, *p* < 0.01).

We further analyzed the contents of amino acids that are related to flesh quality. Glutamic acid is an umami amino acid for pleasing taste [24], while taurine has strong antioxidant properties positively affecting flesh quality [25]. Compared with the control, the contents of amino acids were significantly reduced at 20 and 30 days post-treatment (Figure 7a). Strikingly, a gradual and significant increase in glutamic acid was observed between 20 and 40 days post-treatment (Figure 7b). Moreover, the contents of taurine displayed a significant increase at 20 and 40 days post-treatment, although there was no significant change at 30 days post-treatment (Figure 7c). Therefore, depuration and starvation treatment could improve the contents of some umami amino acids and essential acids in the muscle of yellow catfish.

#### 3.3.4. Aldehydes and Ketones Metabolites of Yellow Catfish Muscle during Depuration and Starvation

**Figure 8:**
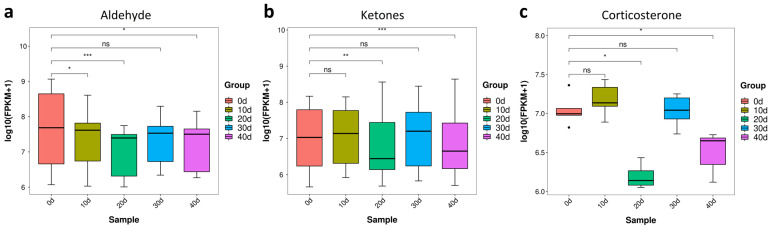
Aldehydes and ketones contents of yellow catfish muscle in five groups. (**a**) Aldehyde; (**b**) Ketones; (**c**) Corticosterone. note: ns, no significance, the *, ** and *** represent significant differences observed between two groups (*p* < 0.05, *p* < 0.01 and *p* < 0.001).

Some aldehydes and ketones are derived from the oxidative deterioration of unsaturated fatty acids (UFAs), which negative affect muscle flavor [9,26]. Corticosterone is released upon stress stimulation in the hypothalamic–pituitary–adrenal axis in animals, which is tightly linked to flesh quality [27,28]. After depuration and starvation treatment, aldehydes showed a significant decrease at 10 days post-treatment (Figure 8a), while ketones displayed a significant decrease at 20 days post-treatment (Figure 8b). The content of corticosterone was greatly reduced at 20 days post-treatment and also inhibited at 40 days post-treatment (Figure 8c). In summary, the contents of aldehydes, ketones and corticosterone all displayed the most reduction at 20 days post-treatment.

## 4. Discussion

Off-flavor and reduced flesh quality is a noteworthy problem in aquaculture fish, especially in catfish, which result in poor acceptability and reduced market price [6,7,8]. Recently, 40 days of depuration and starvation treatment have been used to improve the muscle quality of some fish species, such as *salmo salar*, grass carp, common carp and *Megalobrama amblycephala* [10,11,15,29]. Although depuration and starvation had a positive effect on flesh quality, it can also reduce the body weight of fish. In this study, we tried to investigate the optimal time of depuration and starvation treatment for the improvement of flesh quality in aquaculture yellow catfish. Finally, we found that 20 days of treatment could significantly improve the flesh quality of yellow catfish by reducing excessive fat deposition and changing the contents of muscle metabolites.

Previous studies revealed that the metabolic condition of fish revealed a decrease in the utilization of protein and lipids during depuration and starvation [30]. In this study, the number of unique metabolites in 20 d vs. 0 d was slightly higher than in the other three comparison groups (10 d vs. 0 d, 30 d vs. 0 d, and 40 d vs. 0 d), suggesting that 20 days of treatment could greatly affect muscle metabolism in yellow catfish. Combined with the identification of different metabolites, the results showed that depuration and starvation mainly affected amino acid metabolism, fat metabolism and purine metabolism in the muscle of yellow catfish. Glutamate and aspartic acid were known to be very important for protein mobilization [31]. Fatty acids were important products of fat metabolism [32]. For depuration and starvation, both protein mobilization and fat oxidation were equally used as energy sources from 0-day to 20-day treatment, while fat oxidation was the mainly energy source during 20-day to 40-day treatment, until consumption was to a minimum. The energy source shifted to protein mobilization during the last 10 days of depuration [10]. In the present study, the contents of SFA, UFA, and amino acids in the muscle were not significantly changed at 10 days post-treatment, but they significantly decreased at 20 days post-treatment. Our study shows that protein mobilization and fat oxidation might be the main sources of energy during the first 10 days of depuration in yellow catfish. Subsequently, fat oxidation and amino acid metabolism gradually served as the major energy source during 20 to 40 days post-treatment. Furthermore, histamine could have a serious negative effect on fish muscle quality, and an excessive content of histamine can cause poisoning in humans [33,34]. The content of histamine in yellow catfish muscle decreased with the extension of depuration and starvation time. Moreover, the above results indicated that depuration and starvation could enhance the muscle quality of yellow catfish.

The evaluation of fish flesh quality includes taste, nutritional value and flavor [5]. Glutamic acid is an umami amino acid in fish muscle, and its increased content was beneficial to enhance the freshness of fish meat [24]. In this study, the depuration and starvation treatment enhanced the concentration of glutamic acid in yellow catfish muscle, which was similar with the results in other fish [35,36,37]. It indicated that the depuration and starvation treatment could significantly alter the glutamate metabolism in fish. Previous studies have shown that the ATP is sequentially broken down into ADP, AMP, and IMP in purine metabolism, the IMP production controls the freshness [38], and the AMP production controls the sweet and salty of fish muscle [39]. In the present study, we found that the concentrations of ADP, AMP and IMP were all significantly increased at 20 days post-treatment. The depuration and starvation treatment contributed to the accumulation of fresh-tasting AMP at 20 days post-treatment, and it might also inhibit the degradation of AMP, which was related to the further decomposition of AMP to IMP in yellow catfish. As an active substance regulating the normal physiological activities of the body, taurine is widely distributed in animal tissues and can prevent cardiovascular diseases and enhance immunity [25,40]. Our study showed that the concentration of taurine in yellow catfish muscle increased at 20 days post-treatment, which indicated that the depuration and starvation treatment could improve the content of taurine. To summarize, depuration and starvation could improve the taste and nutritive value in the muscle of yellow catfish.

Excessive fat deposition has a negative effect on the flesh quality of fish [22], while high concentrations of aldehydes and ketones are usually associated with a high level of off-flavors [9], and corticosterone presents a bitter taste [41]. Fat oxidation could reduce fish immunity and flesh quality; especially, a high content of UFAs was easily oxidized [42,43]. After depuration and starvation, the condition factor (CF) and mesenteric fat index (MFI) were significantly decreased at 10 and 20 days post-treatment. Meanwhile, the content of SFAs, UFAs, aldehydes and ketones was significantly reduced at 20 days post-treatment. Since the UFAs were the precursors of fatty aldehydes and fatty ketones in fish [26], the concentration of aldehyde and ketone decreased due to the decreasing of the UFAs during the depuration and starvation. In summary, depuration and starvation treatment for around 20 days can alter the muscle metabolism of yellow catfish by improving the muscle taste and flavor in yellow catfish. Overall, the achievement of this study gives some reference to the theory of green and healthy culture of yellow catfish as well as a program of instruction for the fishery industry.

## Figures and Tables

**Table 1:** Other different metabolites in yellow catfish muscle during depuration. Note, data were calculated as the ratio between depurated samples and the control samples. Different metabolites were screened out according to VIP values (VIP > 1.5) in the OPLS-DA models and *p* value in the *t*-test (*p* < 0.05).

No.	Retention Time (Min)	Identification of Metabolites	MW (Da)	Relative Amount			
				10 d/0 d	20 d/0 d	30 d/0 d	40 d/0 d
1	1.31	1-{4-[2-nitro-4-(trifluoromethyl) phenyl] piperazino} ethan-1-one	339.08	1.80	1.80	1.52	1.65
2	5.30	11-Ketoetiocholanolone	304.20	1.01	0.15	0.48	0.24
3	5.77	13Z,16Z-Docosadienoic acid	336.30	0.63	0.55	0.56	0.55
4	5.22	17alpha-Hydroxyprogesterone	330.22	1.38	0.09	0.94	0.23
5	1.49	2′-Deoxyadenosine	273.08	0.44	0.26	0.30	0.31
6	5.28	3,4-dihydro-2H,6H- [1,3] thiazino [2,3-b] quinazolin-6-one	218.05	0.81	2.30	7.84	1.32
7	5.02	4-Methyl-2-pentanone	100.09	1.69	1.22	1.09	1.02
8	1.33	5-Methoxyindole-3-Carbaldehyde	175.06	1.13	0.51	1.43	0.76
9	1.95	7-{[(2E)-3,7-dimethylocta-2,6-dien-1-yl] oxy}-2H-chromen-2-one	320.14	1.07	0.71	0.79	0.68
10	1.47	AMP	347.06	0.99	12.02	3.67	4.00
11	1.57	ADP	427.03	0.71	4.16	1.58	2.78
12	1.28	Adenylyl sulfate	427.02	1.02	1.56	0.88	1.23
13	1.55	ADP-ribose	559.07	1.45	14.93	4.03	1.94
14	5.15	Aldosterone	406.20	3.87	0.67	2.30	0.84
15	5.52	Arachidonic acid	304.24	0.47	0.14	0.44	0.31
16	5.16	Corticosterone	392.22	1.36	0.14	0.97	0.30
17	1.34	Cytidine	243.09	0.33	0.21	0.24	0.26
18	5.53	DHA	328.24	1.46	0.25	1.06	0.36
19	5.73	Docosatrienoic acid	334.29	0.59	0.30	0.49	0.39
20	5.57	EPA	302.22	0.73	0.22	0.53	0.40
21	5.69	11Z,14Z,17Z-Eicosatrienoic acid	306.26	1.32	0.11	0.90	0.28
22	5.60	8Z,11Z,14Z-Eicosatrienoic acid	306.26	0.78	0.23	0.56	0.31
23	1.46	Erythronolactone	118.03	1.41	4.67	2.80	2.78
24	2.00	Guanosine 5′-diphosphate	443.02	6.79	8.32	3.57	18.48
25	1.65	Guanosine	283.09	0.36	0.40	0.34	0.40
26	1.51	Inosine 5′-diphosphate	111.08	1.28	1.65	1.53	1.66
27	1.56	Inosine	136.04	1.12	0.98	1.07	1.15
28	1.61	IMP	428.01	1.32	2.12	1.83	2.03
29	5.57	Isophorone	268.08	0.79	0.71	0.76	0.75
30	1.45	Methionine	348.05	0.83	0.57	0.45	0.68
31	1.24	Arginine	138.10	0.35	0.33	0.21	0.47
32	1.39	Aspartic acid	149.05	0.72	1.05	1.13	2.27
33	1.53	Glutamic acid	174.11	0.33	0.33	0.31	1.03
34	1.26	Lysine	133.04	0.69	0.45	0.21	0.32
35	1.39	Methionine sulfone	147.05	0.32	0.43	0.29	0.42
36	1.63	Phenylalanine	146.11	0.34	0.32	0.41	0.42
37	1.35	Threonine	181.04	0.74	0.73	0.47	0.63
38	5.57	N-Octyl-2-pyrrolidone	148.05	0.75	1.08	0.88	1.04
39	2.66	o-Veratraldehyde	119.06	0.23	0.04	0.13	0.11
40	5.61	C 16:0	197.18	0.89	0.68	0.67	0.63
41	5.47	Palmitoleic acid	166.06	0.44	0.34	0.20	0.33
42	1.54	Pantothenic acid	256.24	1.02	0.97	0.92	0.87
43	5.25	Pentadecanoic acid	254.22	0.31	0.33	0.30	0.37
44	1.51	p-Hydroxybenzaldehyde	219.11	0.51	0.45	0.57	0.61
45	5.51	Pregnenolone	288.23	0.98	0.36	0.76	0.39
46	5.30	Progesterone	122.04	1.09	0.09	0.64	0.27
47	1.43	Proline	316.24	0.35	0.16	0.29	0.29
48	1.68	Reduced nicotinamide adenine dinucleotide	314.22	2.49	0.72	1.36	0.63
49	1.17	S-adenosylmethionine	115.06	2.14	1.68	1.45	1.03
50	4.90	Sebacic acid	665.13	0.84	1.63	1.11	1.57
51	5.77	C 18:0	398.14	0.61	0.60	0.40	0.64
52	1.37	Taurine	202.12	0.93	1.47	0.98	1.39
53	1.32	UDP-galactose	284.27	1.20	1.19	1.25	0.80
54	1.71	UDP-N-acetyl-alpha-D-glucosamine	125.01	0.71	2.02	0.75	1.70
55	1.43	Uridine	566.05	0.29	0.30	0.23	0.46
56	1.57	Uridine diphosphate glucose	607.08	2.11	14.34	10.98	16.75
57	1.50	Uridine monophosphate	244.07	1.67	1.46	1.28	1.33
58	1.31	Histamine	566.05	0.98	0.35	0.18	0.20

## Data Availability

Data are available in the website https://ngdc.cncb.ac.cn/omix/release/OMIX005160 (accessed on 30 October 2023).
